# Complete plastid genome of *Cheirostylis chinensis* (Orchidaceae, Goodyerinae)

**DOI:** 10.1080/23802359.2019.1698347

**Published:** 2019-12-11

**Authors:** Xiong-De Tu, Sai Zhang, Ding-Kun Liu, Ming-He Li

**Affiliations:** a Key Laboratory of National Forestry and Grassland Administration for Orchid Conservation and Utilization at College of Landscape Architecture, Fujian Agriculture and Forestry University, Fuzhou, China;; b Fujian Colleges and Universities Engineering Research Institute of Conservation and Utilization of Natural Bioresources at College of Forestry, Fujian Agriculture and Forestry University, Fuzhou, China

**Keywords:** Chloroplast genome, Cranichideae, Orchidoideae, phylogeny

## Abstract

The first complete plastid genome of *Cheirostylis*, *Ch. chinensis*, was assembled and analyzed in this study. The total genome was 147,218 bp in length, consisting of a large single-copy region (LSC) of 81,081 bp, a small single-copy region (SSC) of 14,769 bp, and two inverted repeat regions (IRA and IRB) of 25,684 bp. The genome contained 131 genes, including 38 transfer RNA (tRNA) genes, 8 ribosomal RNA (rRNA) genes and 85 protein-coding genes. Phylogenomic analysis indicated that *Ch. chinensis* nested within Goodyerinae.

## Introduction

The genus *Cheirostylis*, belonging to the subtribe Goodyerinae (Orchidaceae, Orchidoideae, Cranichideae), is characterized by the fleshy and moniliform rhizome with internodal rhizoids, flowers with obconical ovaries, connate sepals, a lip containing parallel seriate appendages, and a column with two separate stigma lobes that bear sterile extensions (Pridgeon et al. 2003; Chen et al. 2019). The genus consists of approximately 55 species well represented in tropical Africa, Asia and the Pacific Islands (Govaerts et al. 2012).

In this study, a complete plastid genome of *Cheirostylis*, *Ch. chinensis*, was assembled and annotated. The complete genomic DNA was extracted from fresh leaves using a modified Cetyltrimethylammonium Ammonium Bromide (CTAB) method (Doyle and Doyle 1987) and sequenced by the BGISEQ-500 platform. The samples were collected from Jinan district, Fujian, China (26°38′N, 119°27′E) and the voucher specimen deposited at Herbarium of Fujian Agriculture and Forestry University (specimen code FJFC 0411). The clean reads were used to assemble the complete chloroplast genome by the GetOrganelle pipe-line (Jin et al. 2018), with the chloroplast genome of *Goodyera procera* (GenBank accession KT886429) as the reference sequences. The assembled chloroplast genome was annotated using the Geneious R11.15 (Kearse et al. 2012).

The total chloroplast genome of *Ch. chinensis* (GenBank accession MN641483) was 147,218 bp in length, consisting of a large single-copy region (LSC) of 81,081 bp, a small single-copy region (SSC) of 14,769 bp, and two inverted repeat regions (IRA and IRB) of 25,684 bp. The GC content of the chloroplast genome was 36.79%, while the corresponding values of the LSC, SSC, and IR regions are 34.32%, 29.00%, and 42.93%, respectively. The complete chloroplast genome contains 131 genes, including 38 transfer RNA (tRNA) genes, 8 ribosomal RNA (rRNA) genes and 85 protein-coding genes.

**Figure 1.**
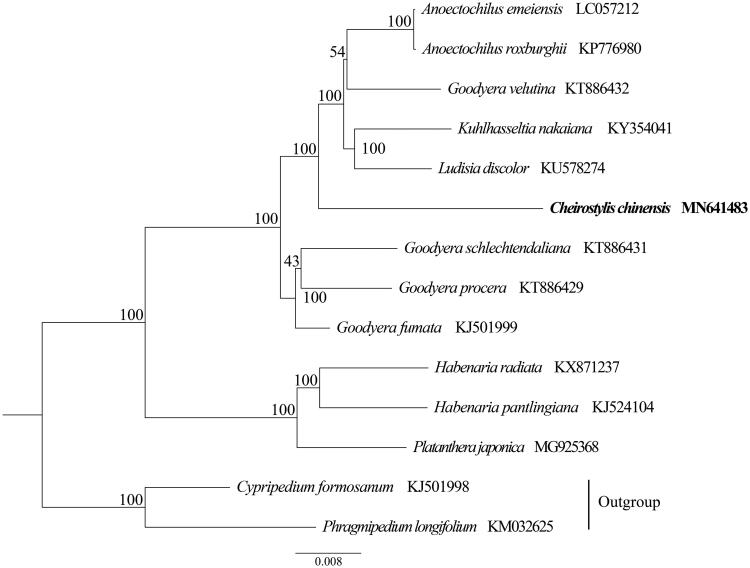
The maximum-likelihood (ML) tree based on the 12 representative plastid genome sequences of Orchidoideae and two outgroup species of Cypripedioideae. The bootstrap value near each node.

To confirm the phylogenetic position of *Ch. chinensis*, a phylogenetic analysis was performed based on 12 species of Orchidoideae and two outgroup species of Cypripedioideae. All sequences were aligned with the HomBlock pipeline (Bi et al. 2018), and the phylogenetic tree constructed by RAxML (Stamatakis 2014) with 1000 ultrafast bootstrap (UFBoot) replicates (Minh et al. 2013; Chernomor et al. 2016). The results showed that *Ch. chinensis* nested within Goodyerinae (Figure 1).
